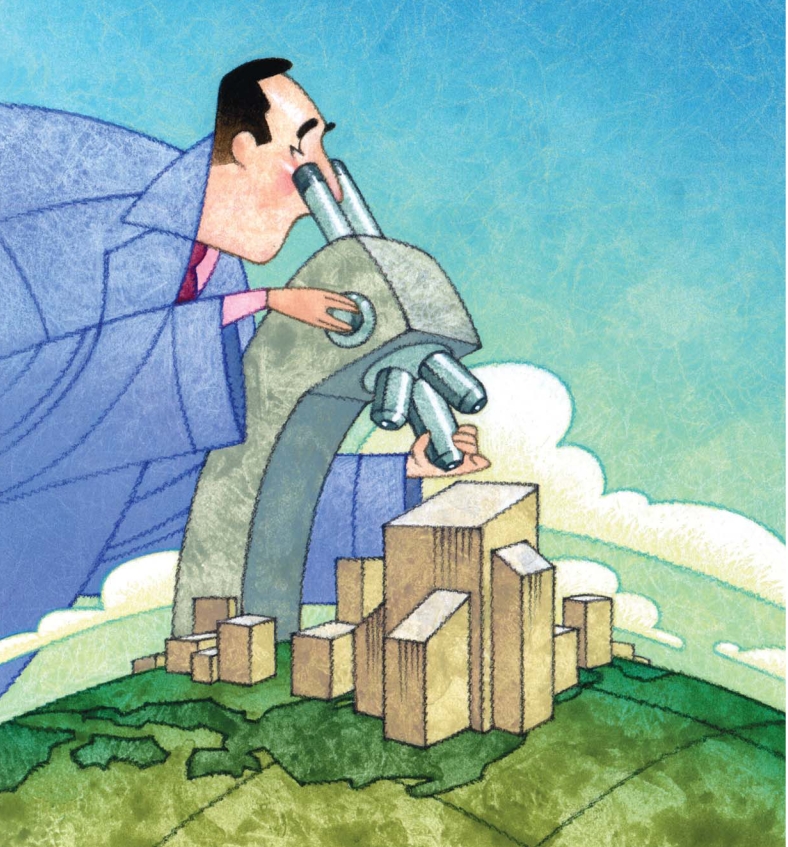# Oversight Without Obstruction: The Challenge for High-Containment Labs

**DOI:** 10.1289/ehp.116-a486

**Published:** 2008-11

**Authors:** John Manuel

A cross the nation, government agencies, industries, and academic institutions are building high-containment biological laboratories to research hazardous pathogens that might accidentally or intentionally be introduced to the United States. Experts agree that the knowledge gained from such labs is a boon to public health—these are the places where the diagnostic test for Ebola virus, antitoxins for botulism, and therapies for multidrug-resistant tuberculosis were developed. But many of these same experts also point to the need for strict oversight of high-containment labs, and some worry that unchecked proliferation of such facilities increases the odds that study pathogens will make their way into the environment. The challenge for governing agencies is to provide researchers the flexibility to do their work while ensuring public safety.

In 2003, the Senate Committee on Governmental Affairs convened a hearing on the potential vulnerabilities of the U.S. food supply and agricultural sector to deliberate contamination. Two years earlier, the United Kingdom had experienced an epidemic of foot-and-mouth disease (FMD) that devastated the British livestock industry. The disease was not introduced intentionally, but many found the timing disquieting, given the subsequent events of 9/11 and deaths later that fall from anthrax sent through the mail. The U.S. agricultural sector accounts for 13% of the gross domestic product and is vulnerable to both intentional and accidental introduction of infectious disease, according to Lawrence Dyckman, director of natural resources and environment for the U.S. Government Accountability Office (GAO). In testimony at the 2003 hearing, Dyckman identified more than 40 contagious foreign animal diseases as threats to the U.S. agricultural economy.

This vulnerability prompted President George W. Bush to issue Homeland Security Presidential Directive 9 in 2004. Among other provisions, the directive called for the secretaries of the U.S. Department of Agriculture (USDA) and Department of Homeland Security (DHS) to develop secure, state-of-the-art biocontainment laboratories to research and develop diagnostic capabilities for exotic animal diseases, including “zoonotic” diseases that can be transmitted to humans.

Three years later, the House Energy and Commerce Subcommittee on Oversight and Investigations held a hearing on the proliferation and oversight of high-containment labs. In testimony before the subcommittee, Gigi Kwik Gronvall, a senior associate with the Center for Biosecurity of the University of Pittsburgh Medical Center, said, “Protecting the nation against destabilizing large-scale epidemics, whether natural or man-made, is an urgent priority. . . . [N]ew high-containment laboratories are necessary if we are to produce the scientific advances needed to develop medical countermeasures against bioweapons and emerging diseases.” At the same time, said Gronvall and other witnesses, the need for rigorous oversight and training of personnel is paramount.

## NBAF in the News

In response to Directive 9, DHS has announced plans to build a National Bio- and Agro-Defense Facility (NBAF), a secure facility being planned by DHS that will support collaborative efforts among researchers from government agencies and academia. NBAF will assume the research responsibilities of the current Plum Island Animal Disease Center (PIADC), located off the tip of Long Island, New York, where most U.S. research on exotic livestock disease has been carried out for the last several decades. The PIADC, constructed in the 1950s, is nearing the end of its life cycle and is not easily accessible to visiting researchers.

DHS is considering six possible sites for the 520,000-square-foot NBAF. These include Plum Island; Athens, Georgia; Manhattan, Kansas; Flora, Mississippi; Butner, North Carolina; and San Antonio, Texas. If built on Plum Island, the NBAF would replace the current PIADC. The Athens site would be located on land owned by the University of Georgia. The Manhattan site would be on the campus of Kansas State University immediately adjacent to the Biosecurity Research Institute. In Mississippi, NBAF would be located at the Flora Industrial Park owned by the Madison County Economic Development Authority. In Butner, NBAF would be located on the Umstead Research Farm owned by the USDA. In San Antonio, NBAF would be located in Texas Research Park, which is owned and developed by the Texas Research & Technology Foundation.

As described by DHS in its draft environmental impact statement released in June 2008, NBAF would cost approximately $45 million to build and employ between 250 and 350 people. Construction would begin in early 2010 and take approximately four years to complete. NBAF would be jointly owned by DHS and the USDA and would be operated by them or by an outside contractor.

NBAF would contain BSL-2, -3, -3Ag, and -4 labs. BSL, short for “biosafety level,” refers to the potential threat posed by the agents studied (see table below). BSL-3 and -4 labs operate under negative air pressure: if the barrier is breached, air flows into rather than out of the room. All exhaust air runs through high-efficiency particulate air filters, with the actual handling of viruses carried out in a 3- by 8-foot biosafety cabinet. At a BSL-3Ag facility, where large animals are being researched, the room itself functions as the primary containment area. Workers handling agents in BSL-3, -3Ag, and -4 labs are required to wear full-body, air-supplied, positive-pressure personnel suits. All critical functions are designed with redundant systems, so for example if the main power is knocked out, back-up generators kick in.

DHS and the USDA have identified six BSL-3 agents for possible study at NBAF (FMD virus, classical swine fever virus, African swine fever virus, Rift Valley fever virus, the *Mycoplasma mycoides* ssp. *mycoides* bacterium that causes contagious bovine pleuropneumonia, and Japanese encephalitis virus) and two BSL-4 pathogens (Nipah virus and Hendra virus). Of these, only Rift Valley fever virus and Nipah virus are zoonotic. Researchers will focus on developing tests to detect the diseases and countermeasures to prevent them. Other pathogens could be studied as need dictates. Scientists will study live and dead animals as well as tick and mosquito vectors.

Although few scientists have questioned the need for NBAF, some question the wisdom of bringing the facility onto the mainland, as would be the case if the laboratory were built anywhere other than Plum Island. The PIADC, by virtue of being situated on an island, is protected by a natural buffer of water that disease vectors and pathogens are unlikely to cross in the event of an accidental release.

In its 1,000-page draft environmental impact statement, DHS categorized potential benefits of the NBAF—which include new biologic knowledge, added jobs, and enhanced health and safety of the population—as “significant.” Under normal operating conditions, the potential impacts at each of the proposed locations—such as noise, traffic, and waste management—were judged to be “negligible,” “minor,” or “moderate.”

**Table:** Biosafety Levels Explained

**BSL-1** facilities are suitable for studying well-characterized agents, such as *Bacillus subtilis*, that are not known to consistently cause disease in healthy adults.
**BSL-2** labs are designed for handling indigenous agents of moderate risk to humans and the environment. Examples include the hepatitis B virus and *Salmonella* bacteria.
**BSL-3** facilities are appropriate for handling pathogens of exotic or indigenous origin with a known potential for serious disease or death resulting from aerosol transmission. *Mycobacterium tuberculosis*, which causes tuberculosis, is a BSL-3 pathogen.
**BSL-3Ag** refers to research with BSL-3 pathogens that primarily affect livestock, although with some diseases, such as Rift Valley fever (RVF), human transmission also is possible.
**BSL-4** facilities are designed to handle exotic pathogens that pose a high risk of life-threatening disease in humans and animals through airborne transmission and for which there is no known vaccine or therapy. Marburg virus is one such pathogen.

**Source:** U.S. Department of Health and Human Services. 2007. Biosafety in biomedical and microbiological laboratories. 5th edition. Washington, DC: U.S. Government Printing Office. Available: http://bmbl.od.nih.gov/.

The impacts of an accidental pathogen release from NBAF, outlined in Appendix D of the report, could be far more serious, potentially resulting in “significant economic impacts” if commercial livestock were exposed, if the pathogen were to infect wild game, or if wildlife were to become endemic reservoirs of disease. The report cites the 2001 outbreak of FMD in the United Kingdom as resulting in the slaughter of more than 6 million animals with US$5 billion lost in tourism, food, and agriculture. The draft environmental impact statement estimates that costs for a similar U.S. outbreak of FMD could reach $30 billion. According to the document, “any country experiencing an outbreak [of FMD] would be subject to a total ban on its exports, suggesting eradication by slaughter may be necessary to regain a trading status.”

DHS estimates, however, that the probability of such an accidental release occurring within the lifetime of the facility is “unlikely but possible” or altogether “unlikely.” This estimate was based on a hazard evaluation/accident analysis model that considered all possible accident initiators and failure modes. Daniel Mead, an assistant research scientist with the Southeast Cooperative Wildlife Disease Study at the University of Georgia College of Veterinary Medicine, says, “I have worked in the lab at Plum Island, and the likelihood of an [infected] insect escaping is next to none. There are so many checks and balances, it’s amazing we get any work done.”

He adds, “Destruction of [infected] animals is pretty common at BSL-3Ag labs. Incineration and chemical destruction are tried-and-true methods. There’s no way in the world a pathogen is going to survive those processes.”

But even the remote prospect of such an accident has led to strong public opposition to NBAF at some of the proposed sites. At a 29 July 2008 public hearing in Butner, speakers were uniformly opposed to NBAF. They also expressed concerns about the projected 25–30 million gallons of wastewater that would be pretreated and sent through the municipal sewer system each year; the increased air emissions from power generators, traffic, and incineration of animal carcasses; and the possible accidental release of infected mosquitos, which would have to be treated by aerial spraying of insecticides over a wide area for a protracted period of time. And they raised questions about the long-term day-to-day management of the facility by an as yet unnamed entity.

Bill Felber, executive editor for the daily *Manhattan* (Kansas) *Mercury*, has covered the public hearings at all six potential NBAF sites. Felber says the Butner speakers were by far the most negative toward NBAF. “The Plum Island crowd was also negative, but more subdued, largely because they don’t think they are being seriously considered [as a site],” he adds. “Georgia had the most debate back and forth, both positive and negative. Manhattan was about three to one in favor of the facility. The Mississippi and Texas speakers were uniformly positive.” DHS officials have not said which site they prefer. A final environmental impact statement is due by December 2008, to be followed by a decision on the site.

## Laboratory Expansion

The debate over NBAF mirrors a larger debate that is arising over the necessity and wisdom of constructing multiple high-containment labs across the United States. In 2007 the House Subcommittee on Oversight and Investigations asked the GAO to investigate the proliferation of BSL-3 and -4 labs, identify the federal agencies responsible for monitoring these labs, and analyze recent incidents at three high-containment labs. In the 2007 report *High-Containment Biosafety Laboratories: Preliminary Observations on the Oversight of the Proliferation of BSL-3 and BSL-4 Laboratories in the United States*, the GAO reported there were only two BSL-4 labs operating in the United States prior to 1990—the U.S. Army Research Institute of Infectious Diseases in Fort Detrick, Maryland, and the Centers for Disease Control and Prevention (CDC) lab in Atlanta, Georgia. Between 1990 and 2000, three new BSL-4 labs were built—one at the Viral Immunology Center at Georgia State University, one at the National Institutes of Health in Bethesda, Maryland, and one at the private Southwest Foundation for Biomedical Research in San Antonio. Since 9/11, 10 additional BSL-4 labs have been built, are under construction, or are in the planning stages.

The proliferation of BSL-3 labs has been even greater. The GAO reported that the expansion of BSL-3 labs is widespread across the country. Most state governments now have some BSL-3 capacity, at least for diagnostic and analytical services, because of the need for individual state response to bioterrorist threats.

Expansion is also taking place within the academic community. As part of its Strategic Plan for Biodefense Research, the National Institute of Allergy and Infectious Diseases (NIAID) has funded 13 Regional Biocontainment Laboratories to provide regional BSL-3 capacity for academic research. In H.R. 7041, legislation introduced to Congress on 24 September 2008, Representative Christopher Carney (D–PA) requested an additional $71 million over the next three years to fund “surge capacity” at selected Regional Biocontainment Laboratories in the event of a large-scale terror event.

“Recent natural and bioterrorist events involving infectious agents have made it very clear that from a strategic national perspective, a serious shortage of BSL-3 and BSL-4 laboratory space exists,” the NIAID stated in “The Need for Biosafety Laboratory Facilities,” a fact sheet posted on the institute’s website. “Many U.S. institutions and companies with infectious disease programs have BSL-3 laboratories required to perform their research. Most such laboratories, however, are small, dedicated to particular uses, or in need of modernization.”

The GAO report counted 1,356 BSL-3 labs registered with the CDC and USDA Select Agent Programs. These programs regulate the possession, use, and transfer of certain biological agents that could pose “a severe threat to public health and safety.” The CDC program governs agents that pose a threat to human health, whereas the USDA program governs agents that pose a threat to plant or animal health; some agents, such as anthrax, affect both humans and animals and are thus regulated by both agencies. Any BSL-3 or -4 facility that handles select agents must register with the appropriate Select Agent Program and demonstrate that it meets federal safety requirements for working with those agents. However, not all BSL-3 and -4 pathogens are designated as select agents. And the number of high-containment labs not registered with the CDC or USDA is unknown.

The GAO’s concern, as expressed in the 2007 report, is that no single federal agency is responsible for tracking the number of all BSL-3 and -4 labs in the United States or the attendant risks associated with the proliferation of such labs. Yet, federal agencies charged with protecting the public health need to know where these facilities are located and what agents are handled there if they are to fulfill their missions. “According to [academic experts in microbiological research], there is a baseline risk associated with any high-containment lab, attributable to human errors,” the report stated. “With expansion, the aggregate risks will increase.”

GAO cites three recent incidents as evidence of the need for better reporting, design, and maintenance of biocontainment labs. In 2006, a lab worker at Texas A&M University was exposed to *Brucella*, a BSL-3 pathogen, and later contracted brucellosis. The worker was not authorized or trained to work with that bacterium. Confirmation of the disease was not made until 62 days after exposure, and Texas A&M officials did not report the incident to the CDC as required by law.

In June 2007, the CDC campus in Atlanta was struck by lightning, knocking out both primary and back-up power to its BSL-4 facility. Although no live agents were in the facility at the time, the outage shut down the negative air pressure system that is key to keeping dangerous agents from escaping the containment area.

A third incident, also in 2007, involved the spread of FMD virus to several farms near Pirbright, United Kingdom, the site of several high-containment labs that work with that pathogen. Though the definitive cause of the release has not been determined, officials suspect that contaminated waste-water from the Pirbright labs leaked into the surrounding soil, and the live virus was carried offsite by vehicles splashed with contaminated mud.

Given the real need for high-containment facilities, what measures can be instituted to ensure safety? “We are confident that the procedures we have in place have improved safety and security for [select agent] labs,” says Richard Besser, director of the Coordinating Office for Terrorism Preparedness and Emergency Response at the CDC. At the same time, Besser acknowledges some are concerned about safety and security in labs that handle dangerous pathogens not covered by the select agent rules.

Richard Ebright, lab director at the Waksman Institute of Microbiology at Rutgers University, believes policies at high-containment labs should include full video surveillance at the entrance and in the labs, having two persons present when handling a virus, psychological testing for employees, and unannounced inspections in work areas and after leaving the lab.

In her 2007 testimony, Gronvall pointed to the need for expanded training in biosafety for researchers working in high-containment labs, as well as a greater number of biosafety officers who can help researchers determine the safest procedures and practices on an experiment-by-experiment basis. Systematic analysis of safety and operational issues is critical, she wrote, if labs are to learn from their own and others’ mistakes. She suggested looking to the aviation, nuclear, and chemical industries for possible reporting models that might allow laboratories to share experiences and avoid future accidents.

Besser agrees, with a caveat. “It’s important to continually review our systems, to ask whether there are things that should be done in other labs to ensure safety and security,” he says. “But it’s also important to ensure that any new regulations will have a high likelihood of improving safety and security and not restrict research unnecessarily.”

In response to concerns expressed by government and scientific experts, the Department of Health and Human Services, USDA, DHS, and Department of Defense have formed a task force to look at lab safety and develop a set of recommendations for improvement. The report is due this fall.

## Figures and Tables

**Figure:**